# Rapidly Progressive Emphysematous Pancreatitis With Massive Hemorrhage and Multi-Organ Failure: A Severe Sequela of COVID-19

**DOI:** 10.7759/cureus.33717

**Published:** 2023-01-12

**Authors:** Raghav Bassi, Hamza Alzghoul, Kipson Charles, Ariel Ruiz de Villa, Charles R Russell, Peters Okonoboh

**Affiliations:** 1 Internal Medicine, University of Central Florida College of Medicine, Graduate Medical Education/North Florida Regional Medical Center, Gainesville, USA; 2 Internal Medicine, HCA Florida North Florida Hospital, Gainesville, USA; 3 Internal Medicine, University of Central Florida, School of Medicine/North Florida Regional Medical Center, Gainesville, USA; 4 Internal Medicine - Critical Care, University of Central Florida, School of Medicine/North Florida Regional Medical Center, Gainesville, USA

**Keywords:** multiorgan system failure, severe sepsis, hemorrhagic pancreatitis, acute respiratory distress syndrome [ards], coronavirus disease (covid-19), acute necrotizing pancreatitis

## Abstract

The COVID-19 global pandemic continues to wreak havoc on a number of affected patients and poses a significant burden on the healthcare system. Even though it has been over two years since the pandemic emerged, clinical presentations in affected patients continue to appall clinicians. Emphysematous pancreatitis is a rare, fatal complication of acute necrotizing pancreatitis presenting with a high mortality rate. This rare entity stems from superinfection of acute necrotizing pancreatitis with gram-negative bacteria, most commonly from *Escherichia coli *(*E. coli), *among others. Herein, we present a rare case of acute necrotizing pancreatitis complicated by emphysematous necrosis with hemorrhagic conversion and *E. coli *septicemia in a 60-year-old morbidly obese male patient without any underlying risk factors. He presented with respiratory failure in the setting of COVID-19 and was subsequently diagnosed with acute necrotizing pancreatitis complicated by emphysematous necrosis. To our knowledge, emphysematous pancreatitis in the setting of COVID-19 with no other attributable causes for pancreatitis was not previously reported in the literature. This article aims to report an unusual association between COVID-19 infection and acute emphysematous pancreatitis with evidence of hemorrhagic conversion. Furthermore, given the neoteric nature of this viral infection, we hope to promote sensitivity toward capturing additional clinical features associated with active COVID-19 infection, with the goal to keep clinicians abreast with its many possible sequelae.

## Introduction

Approximately 80% of acute pancreatitis cases in the United States are due to alcohol consumption and cholelithiasis [1-3]. Viral pancreatitis is a rare phenomenon and is scantly reported in the literature, with the most common culprits being hepatitis viruses, measles, mumps, varicella, and cytomegalovirus [4]. Throughout the literature, there have been a few case reports describing COVID-19-associated acute necrotizing pancreatitis [5,6]. Emphysematous pancreatitis, however, is an even more sparse entity and, to our knowledge, was never reported in the literature to be associated with COVID-19 infection. We hope to contribute to our current scientific knowledge about COVID-19 by presenting a case of a 60-year-old male patient diagnosed with COVID-19 infection who then developed rapidly progressive emphysematous pancreatitis and its fatal complications.

## Case presentation

**Figure 1:**
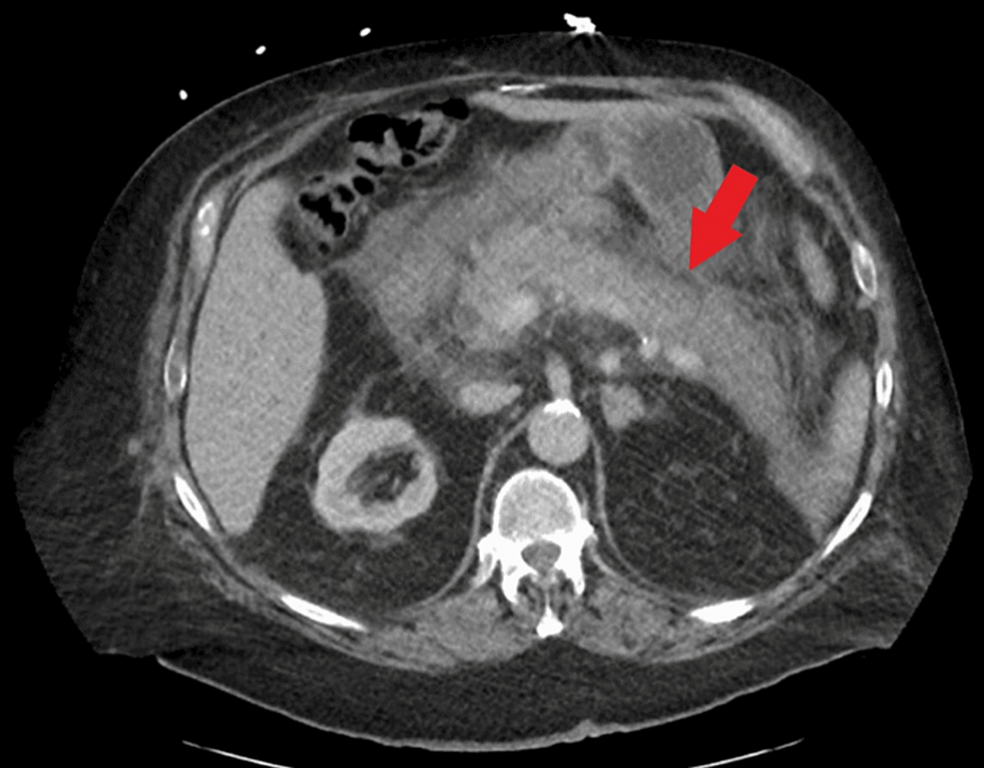
CT of the abdomen and pelvis with contrast revealing extensive peripancreatic inflammatory changes, extending to the adjacent pylorus and duodenum suggestive of moderate-to-severe pancreatitis. There is also notable intrahepatic and extrahepatic biliary ductal dilation.

**Figure 2:**
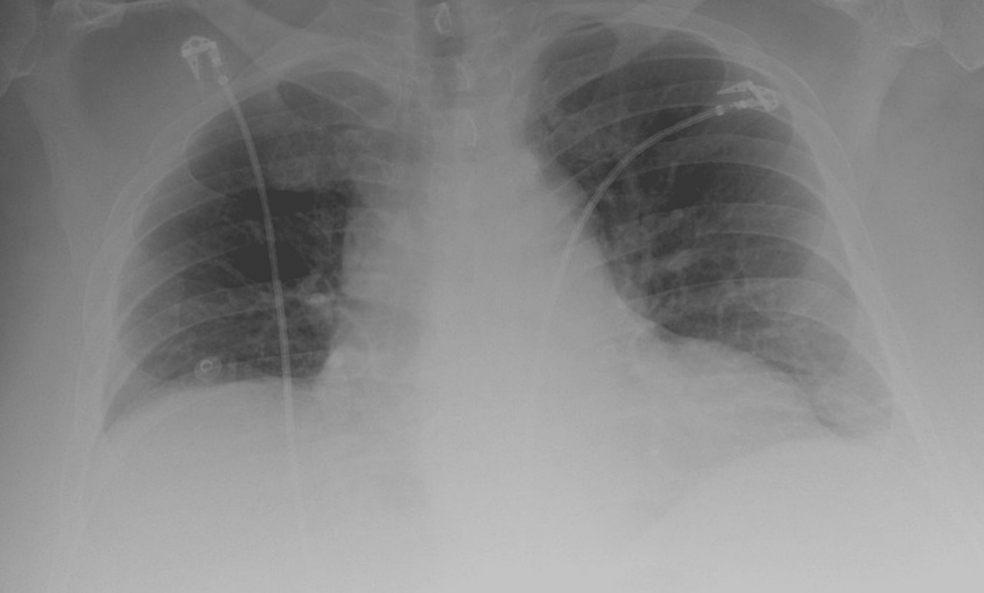
Chest X-ray on presentation revealing bilateral infiltrates suggestive of viral pneumonia with some bibasilar pleural effusions.

A 60-year-old male with a past medical history of class III obesity status post-gastric sleeve surgery, and cholecystectomy, presented to the emergency department with a chief complaint of intractable nausea and vomiting with worsening acute epigastric abdominal pain one day prior to admission. He also developed a non-productive cough and non-bloody diarrhea at around the same time. The patient denied any history of trauma, recent medication changes, chronic diseases, smoking, or alcohol use. On physical examination, he was afebrile, tachycardic, and hypertensive, and had an oxygen saturation of 92% on room air. He had epigastric tenderness with normal bowel sounds and no evidence of guarding, rebound tenderness, or distention. Laboratory investigations were significant for leukocytosis at 13,000 µL and lipase of 9,989 IU/L. Liver function tests revealed transaminemia with aspartate transaminase (AST) of 158 IU/L, alanine transaminase (ALT) of 161 IU/L, total bilirubin of 2.1 mg/dL, and alkaline phosphatase of 520 IU/L. A comprehensive metabolic panel revealed an acute kidney injury with a creatinine of 1.42 mg/dL and normal calcium of 9.5 mg/dL. Lipid panel was within normal limits. The patient was also tested for COVID-19 given his respiratory symptoms and was subsequently found to be positive. Computed tomography (CT) of the abdomen and pelvis with contrast revealed evidence of moderate-to-severe pancreatitis with extensive peripancreatic inflammatory changes, extending to the adjacent pylorus and duodenum. There was also an increased size of the intrahepatic and extrahepatic biliary ducts with a surgically removed gallbladder with no evidence of gallstones (Figure 1). An abdominal Doppler ultrasound revealed good flow, ruling out possible portal vein thrombosis. A chest X-ray revealed bibasilar infiltrates suggestive of underlying COVID-19 pneumonia with some bilateral pleural effusions (Figure 2). The patient was admitted for acute pancreatitis and was started on supportive therapy, bowel rest, and adequate pain control. He was placed on 4 liters/minute of supplemental oxygen through a nasal cannula, and given his increased oxygen requirements, he was started on remdesivir and dexamethasone.

**Figure 3:**
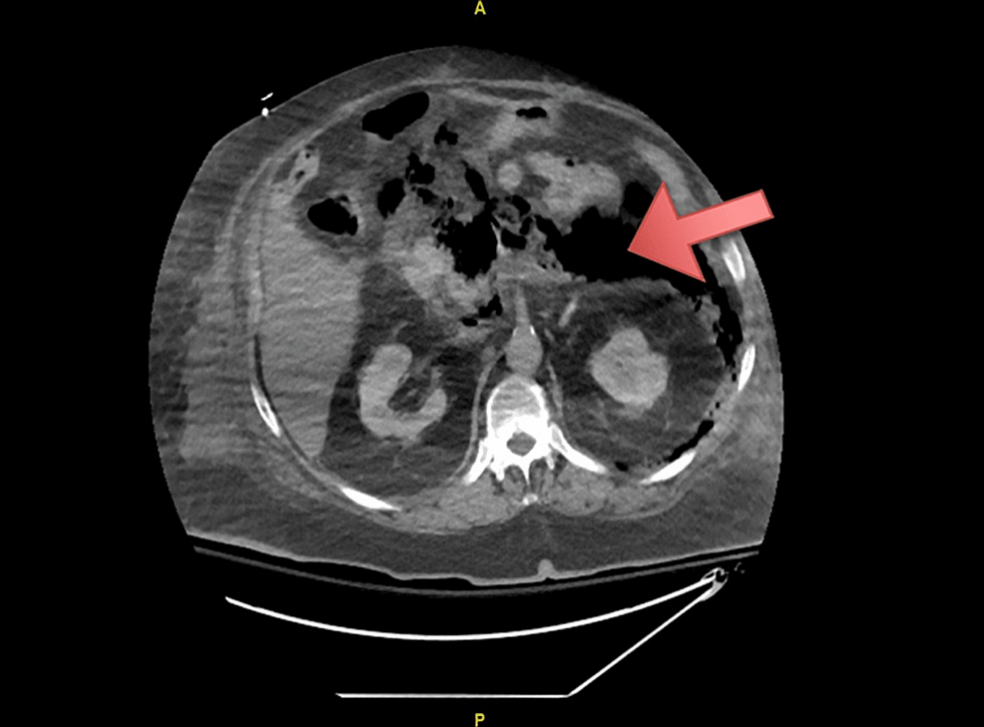
CT of the abdomen and pelvis revealing extensive gas collection in the retroperitoneum extending to the root of the mesentery and left pararenal space consistent with emphysematous pancreatitis.

On hospital day 2, the patient developed acute encephalopathy with progressive respiratory failure requiring 6 liters/minute of supplemental oxygen through a nasal cannula. Morning labs revealed an acute worsening of kidney function and liver function, as summarized in Table 1. Magnetic resonance cholangiopancreatography was planned to rule out choledocholithiasis given dilation of the biliary tracts seen on imaging; however, given the patient’s body habitus, he was unable to fit in the scanner. Interventional radiology (IR) was consulted for a percutaneous cholangiogram; however, the procedure was postponed due to the patient’s progressive respiratory failure. The patient's respiratory failure continued to deteriorate and a rapid response was called due to interval development of hypotension. On arrival, the patient's oxygen saturation was 72%, with associated hypotension. He was placed on a non-rebreather mask and given 1 liter of intravenous fluids. On examination, his abdomen was protuberant and peritonitic, with guarding and rebound tenderness present diffusely throughout his abdomen. The patient was subsequently transferred to the intensive care unit, where he was intubated and placed on mechanical ventilation. Norepinephrine infusion was started for circulatory support. A repeat stat CT of the abdomen and pelvis revealed extensive gas collection in the retroperitoneum that extended to the root of the mesentery and left pararenal space, consistent with emphysematous pancreatitis (Figure 3). Afternoon labs were then redrawn, which revealed multi-organ failure with worsening lactic acidosis, renal function, and liver function, as summarized in Table 1. Blood cultures were collected prior to the administration of empiric antibiotic therapy, and general surgery consultation was placed. The patient was too unstable to be taken to the operating room, and therefore IR was reconsulted for a percutaneous drain placement. A pigtail catheter was inserted into the pancreas with the release of massive intraperitoneal air and copious amounts of brownish red blood. Intraprocedural cultures were collected and also sent to the lab for further investigation. The patient was appropriately managed with vasopressors, intravenous antibiotics, and antiviral therapy for his COVID-19 infection. With the initiation of remdesivir and high-dose steroids, his lipase continued to trend down, as seen in Table 1.

**Table 1:** Patient laboratory values on hospital day 1, day 2, and day 3 as seen above. AST, aspartate transaminase; ALT, alanine transaminase; BUN, blood urea nitrogen; GFR, glomerular filtration rate; MCV, mean corpuscular volume

Labs	Day 1	Day 2 Morning	Day 2 Afternoon	Day 3	Reference Range
White cell count	13	9	3.5	20	4.0-10.5 [10^3 ^µL]
Hemoglobin	16.7	14.7	14.5	13.7	13.7-17.5 [g/dL]
Hematocrit	52.5	44.6	44.6	45.2	40.1-51 [%]
Platelets	238	171	118	98	150-400 [10^3 ^µL]
MCV	94.1	93.3	93.1	99.6	79.0-92.2 [fL]
Sodium	136	134	136	137	136-145 [mmol/L]
Potassium	3.7	4.2	4.6	4.5	3.5-5.1 [mmol/L]
Chloride	98	105	107	106	98-107 [mmol/L]
Carbon dioxide	22	21	20	10	21-32 [meq/L]
Glucose	206	372	298	72	74-106 [mg/dL]
BUN	26	45	53	58	7-18 [mg/dL]
Creatinine	1.42	1.88	2.56	3.75	0.6-1.30 [mg/dL]
GFR	51	37	26	17	0-120 [mL/min]
Calcium	9.5	8.8	8.3	7.5	8.5-10.1 [mg/dL]
Lipase	9989	7972	7664		73-393 [units/L]
Total bilirubin	2.1	8.4	10.2	11.3	0.2-1.0 [mg/dL]
AST	158	114	107	127	15-37 [units/L]
ALT	161	154	127	123	13-56 [units/L]
Alkaline phosphatase	520	445	384	422	46-116 [units/L]
Magnesium		1.8	1.9		1.8-2.4 [mg/dL]
Lactic acid	5.1	10.2	16.6	18.60	0.4-2.0 [mmol/L]

On hospital day 3, the patient’s condition continued to deteriorate as he now required three vasopressors, an intravenous infusion of sodium bicarbonate, and stress dose steroids. Day 3 labs were drawn, which revealed worsening multi-organ dysfunction, as summarized in Table 1. Blood cultures grew *Escherichia coli* (*E. coli*), and empiric antibiotics were de-escalated to intravenous meropenem. Wound cultures came back positive for *Bacteroides fragilis*. Repeat chest X-ray revealed worsening bilateral airspace disease, suggestive of acute respiratory distress syndrome in the setting of COVID-19 and pancreatitis. The palliative team was consulted, and given the patient's rapid deterioration over the prior 24 hours, the family opted to undergo compassionate extubation.

## Discussion

In the United States, around 80% of acute pancreatitis cases are caused by alcohol use and cholelithiasis [1-3]. In extremely rare cases, the etiology of acute pancreatitis can be attributed to a viral origin, with viral hepatitis, measles, mumps, varicella, and cytomegalovirus being the most common culprits [4]. There is insufficient evidence that COVID-19 causes acute pancreatitis; however, there are published case reports describing the occurrence of acute necrotizing pancreatitis in the setting of COVID-19 without any other obvious cause [5,6]. A large retrospective cohort study analyzing 63,822 COVID-19 patients in Spain revealed that there was no significant association in the development of acute pancreatitis in COVID-19 patients, with less than 1% of patients developing this complication [7]. Furthermore, 10% to 30% of all cases of pancreatitis remain idiopathic [2,3]. In a retrospective study conducted by Kumar et al. assessing 985 COVID patients, the etiology of acute pancreatitis remained unclear in almost 30-60% of patients [8]. Therefore, it is reasonable to conclude that the etiology of acute pancreatitis in some of these patients could have been either directly or indirectly caused by COVID-19. The fact that our patient's lipase improved with the initiation of conventional antiviral and steroid therapy for COVID-19 is suggestive of an association between COVID-19 and the subsequent cultivation of pancreatitis. Furthermore, our patient presented with acute hypoxic respiratory failure likely secondary to acute respiratory distress syndrome (ARDS) as per the Berlin criteria, but it is unclear if this was due to his underlying COVID-19 infection or a sequalea of his severe pancreatitis. His respiratory status continued to decline despite a mild improvement in his lipase, suggesting that his ARDS was likely COVID-19 mediated.

Emphysematous pancreatitis is a rare, fatal complication of acute necrotizing pancreatitis with a mortality rate as high as 50% [9]. This rare entity results from superinfection of acute necrotizing pancreatitis with a gram-negative bacteria, most commonly from *E. coli*, as seen in the case above [10]. We present a case of a 60-year-old COVID-19 patient with no reported history of alcohol use or tobacco smoking and a past surgical history of cholecystectomy who presented with signs and symptoms of acute necrotizing pancreatitis complicated by emphysematous necrosis and *E. coli* septicemia. To our knowledge, emphysematous pancreatitis in the setting of COVID-19 with no other attributable causes for pancreatitis has not been previously reported in the literature. Our patient had a history of cholecystectomy 12 years prior to his presentation and did not have any evidence of biliary stones on ultrasound or CT scan. In addition, his lipid panel was within normal limits. Based on our patient’s history and laboratory findings, alcohol-induced, hypertriglyceridemia, and biliary stones as possible etiologies of his patient’s pancreatitis were ruled out. The rapid progression of necrotizing pancreatitis to fulminant emphysematous pancreatitis in under 24 hours makes this case more compelling.

Emphysematous pancreatitis can be further divided into two main subtypes: the first being the aggressive type with acute onset and the second being a less aggressive type with a delayed onset [10]. Some important prognostic factors that indicate a poor outcome include septicemia and hypovolemia from massive hemorrhage, as seen in the case above [10]. Our patient can be classified into the aggressive type given his acute and rapid deterioration with signs of hypovolemia and hemorrhage. These patients can be predisposed to both arterial and venous bleeds given increased space of the emphysematous space [10]. As seen in the case presentation above, pigtail drainage in the pancreas revealed intraperitoneal free air and frank blood output, suggestive of a retroperitoneal bleed. The etiology of our patient's hemodynamic instability was likely multi-factorial and most attributable initially to septic shock with inadequate source control compounded by a component of hypovolemic shock given the massive blood loss and perforation.

COVID-19 is a known hypercoagulable disease increasing the risk of thrombosis and thromboembolism. Initial cases early on in the pandemic have revealed that this infection results in a unique, profoundly prothrombotic milieu causing both venous and arterial thrombosis [11,12]. Proposed hypotheses include a heightened inflammatory response that leads to thromboinflammation through mechanisms such as endotheliitis and cytokine activation [10]. It has also been suggested that the virus itself can possibly activate the coagulation cascade [5]. During this pandemic, clinicians worldwide have witnessed the effects of thromboembolic events in patients infected including deep venous thrombosis, pulmonary embolism, embolic stroke, and bowel ischemia. Given our patient’s presentation and severity of his disease, thrombotic disease was ruled out, with an abdominal Doppler revealing good flow, making the possibility of an embolic event resulting in acute pancreatic ischemia either via micro-embolic or a larger vessel occlusion unlikely. Pancreatic ischemia is a rare cause of acute pancreatitis, and, in most cases, it results in mild or moderate pancreatitis; however, in extremely rare instances, it can progress to necrotizing pancreatitis similar to the events of the case above [5].

A study published by Wang et al. reported the presence of angiotensin-converting enzyme (ACE2) receptors on pancreatic islets and exocrine cells. ACE2 and transmembrane protease serine 2 are thought to play a pivotal role in the pathophysiology of acute pancreatitis by COVID-19 [11]. However, robust data are still lacking, and more research is needed to elucidate the exact mechanism. In 2020, de-Madaria and Capurso applied a Bradford Hill causality criteria framework to examine the causality of pancreatitis in the setting of COVID-19 through autopsy series of COVID-19 patients with and without concomitant clinically evident acute pancreatitis to help reveal the mechanisms behind this possible, but yet unproven, association [12]. Interestingly, COVID-19 was isolated from samples of a pancreatic pseudocyst and pancreatic tissue in patients with acute pancreatitis, suggesting a relationship between COVID-19 and pancreatitis [12].

Lastly, the coexistence of COVID-19 and acute pancreatitis leads to a higher incidence of mortality and multi-organ failure. A study comparing outcomes of acute pancreatitis between patients with and without a positive COVID-19 polymerase chain reaction (PCR) result showed higher morbidity and mortality in patients with positive PCR results, suggesting a deleterious relationship between the two processes [13]. Another study assessing risk factors for mortality in patients with emphysematous pancreatitis concluded that shock was significantly associated with a higher mortality risk [14]. Our patient met the criteria of septic shock and subsequently developed multi-organ failure. Withdrawal of life support was taken by the patient’s surrogate decision-maker after a rapidly progressive course of deterioration. The relationship between COVID-19 and pancreatitis is sparsely reported in the literature. We, therefore, aim to foster further studies of this relationship to raise cognizance of the rapidly detrimental effects of this association, including fulminant emphysematous pancreatitis, multi-organ failure, and ultimately death.

## Conclusions

COVID-19 and its sequelae continue to remain a conundrum due to its unique, novel impact globally and the many different complications it can impose on affected patients. In this article, we herein report one of the first incidences of acute necrotizing pancreatitis with severe hemorrhage as a sequela of active COVID-19 infection to our knowledge. Being cognizant of the various complications of COVID-19 including this highly deleterious sequela should help clinicians in promptly recognizing and managing patients affected by this entity.
